# Supplementary material for: The gene-regulating proteins NONO and SFPQ assemble into ordered filaments
Source: Commun Biol. 2025 Dec 31;9:117. doi: 10.1038/s42003-025-09396-8 (PMC12848012; doi:10.1038/s42003-025-09396-8)
Supplement: Supplementary file 2 — Supplementary Information [file 42003_2025_9396_MOESM2_ESM.pdf]

Supplemental Information

The gene-regulating proteins NONO and SFPQ assemble into ordered filaments

Tim Rasmussen[1,2], Jannik Küspert[3], Lars Schönemann[1], Dietmar Geiger[3,]*, Bettina Böttcher[1,2,]*

[1] University of Würzburg, Rudolf Virchow Centre, 97080 Würzburg, Germany

[2] University of Würzburg, Biocentre, Chair of Biochemistry II, 97074 Würzburg, Germany

[3] University of Würzburg, Julius-von-Sachs-Institute for Biosciences, Department of Molecular Plant Physiology and Biophysics, 97082 Würzburg, Germany

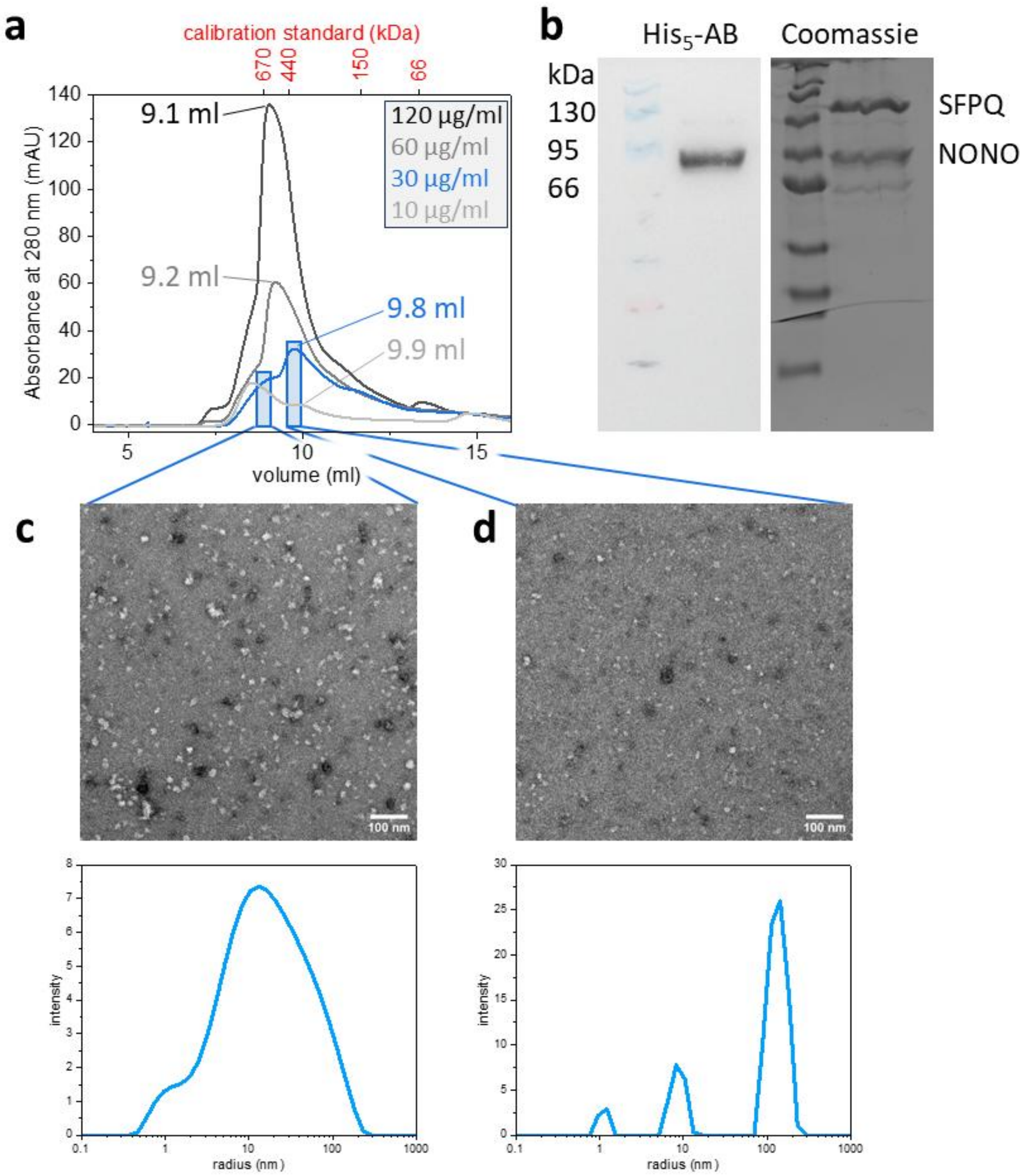

Supplementary Fig. 1: NONO-SFPQ purification. a Size exclusion chromatography of NONO-SFPQ at different concentrations shows a shift of the main peak to larger sizes at higher concentrations. The SEC was performed on a Superdex 200 Increase 10/300. The size calibration is indicated in red. However, the shape and disordered region of NONO/SFPQ do not allow a direct correlation to mass. **b** The SDS-PAGE shows two main bands in the Coomassie-stained gel at about 80 and 110 kDa which were identified by mass spectrometry as NONO and SFPQ, respectively (right). The former showed a positive detection with a His5-antibody on Western blots (left). **c** Shoulder and **d** peak fractions of the 30 µg/ml SEC run (blue) were further analysed by negative stain EM (top) and size distribution determined by dynamic light scattering (bottom), indicating a higher polydispersity in the shoulder.

```
hamster_NONO      1 MQSNKTFNLEKQNHTPRKHHQHHHQQHHQQQQQQQQPPPPIPANGQQASSQNEGLTIDLKNFRKPG   66
human_NONO        1 MQSNKTFNLEKQNHTPRKHHQHHHQQQHHQQQQQQPPPPPIPANGQQASSQNEGLTIDLKNFRKPG   66
cons              1 **************************:*:****** ******************************   66
                                                        RRM1
hamster_NONO     67 EKTFTQRSRLFVGNLPPDITEEEMRKLFEKYGKAGEVFIHKDKGFGFIRLETRTLAEIAKVELDNM  132
human_NONO       67 EKTFTQRSRLFVGNLPPDITEEEMRKLFEKYGKAGEVFIHKDKGFGFIRLETRTLAEIAKVELDNM  132
cons             67 ******************************************************************  132
                                                        RRM2
hamster_NONO    133 PLRGKQLRVRFACHSASLTVRNLPQYVSNELLEEAFSVFGQVERAVVIVDDRGRPSGKGIVEFSGK  198
human_NONO      133 PLRGKQLRVRFACHSASLTVRNLPQYVSNELLEEAFSVFGQVERAVVIVDDRGRPSGKGIVEFSGK  198
cons            133 ******************************************************************  198
                                                                   NOPS
hamster_NONO    199 PAARKALDRCSEGSFLLTTFPRPVTVEPMDQLDDEEGLPEKLVIKNQQFHKEREQPPRFAQPGSFE  264
human_NONO      199 PAARKALDRCSEGSFLLTTFPRPVTVEPMDQLDDEEGLPEKLVIKNQQFHKEREQPPRFAQPGSFE  264
cons            199 ******************************************************************  264
                                                coiled-coil domain
hamster_NONO    265 YEYAMRWKALIEMEKQQQDQVDRNIKEAREKLEMEMEAARHEHQVMLMRQDLMRRQEELRRMEELH  330
human_NONO      265 YEYAMRWKALIEMEKQQQDQVDRNIKEAREKLEMEMEAARHEHQVMLMRQDLMRRQEELRRMEELH  330
cons            265 ******************************************************************  330

hamster_NONO    331 NQEVQKRKQLELRQEEERRRREEEMRRQQEEMMRRQQEGFKGTFPDAREQEIRMGQMAMGGAMGIN  396
human_NONO      331 NQEVQKRKQLELRQEEERRRREEEMRRQQEEMMRRQQEGFKGTFPDAREQEIRMGQMAMGGAMGIN  396
cons            331 ******************************************************************  396

hamster_NONO    397 NRGAMPPAPVPTGTPAPPGPATMMPDGTLGLTPPTTERFGQAATMEGIGAIGGTPPAFNRPAPGAD  462
human_NONO      397 NRGAMPPAPVPAGTPAPPGPATMMPDGTLGLTPPTTERFGQAATMEGIGAIGGTPPAFNRAAPGAE  462
cons            397 ***********:************************************************.****:  462

hamster_NONO    463 FAPNKRRRY  471
human_NONO      463 FAPNKRRRY  471
cons            463 *********  471
```

Supplementary Fig. 2: Sequence alignment of hamster and human NONO. In the consensus row identity is indicated as (*), low similarity as (.), and high similarity as (:). Hamster and human NONO have 99% sequence identity. The histidine-rich motif in NONO is marked in yellow. The alignment was calculated with M-Coffee[1].

```
hamster_SFPQ      1 MSRDRFRSRGGGGGGFHRRGGGGGRGGLHDFRSPPPGMGLNQNRGPMGPGPG--GPKPPIPPPPPH   64
human_SFPQ        1 MSRDRFRSRGGGGGGFHRRGGGGGRGGLHDFRSPPPGMGLNQNRGPMGPGPGQSGPKPPIPPPPPH   66
cons              1 ****************************************************  ************   66

hamster_SFPQ     65 QQQPQQPPPQQPPPQQPPPHQQPPPHQPPHQQ--PPPPPQDSSKPVVPQGPGSAPGVSPAPPPAGS  128
human_SFPQ       67 QQQ-QQPPPQQPPPQQPPPHQ-PPPHPQPHQQQQPPPPPQDSSKPVVAQGPGPAPGVGSAPPASSS  130
cons             67 *** ***************** ****  ****  *************.****.****..***.:.*  132

hamster_SFPQ    129 APPANPPTTGAPPG--PGPTPTPPPAVTSATPGPPPPSTPSSGVSTTPPQSGGPPPPPAGGAGPGP  192
human_SFPQ      131 APPATPPTSGAPPGSGPGPTPTPPPAVTSAPPGAPPPTPPSSGVPTTPPQAGGPPPPPAAVPGPGP  196
cons            133 ****.***:*****  **************.**.***:.*****.*****:********. .****  198

hamster_SFPQ    193 KQGPGPGPGGPKGGKMPGGPKPGGGPGMGAPGGHPKPPHRGGGEPRGGRQHHPPYHQQHHQGPPPG  258
human_SFPQ      197 GPKQGPGPGGPKGGKMPGGPKPGGGPGLSTPGGHPKPPHRGGGEPRGGRQHHPPYHQQHHQGPPPG  262
cons            199     ***********************:.:************************************  264
                                                                              RRM1
hamster_SFPQ    259 GPAARTEEKISDSEGFKANLSLLRRPGEKTYTQRCRLFVGNLPADITEDEFKRLFAKYGEPGEVFI  324
human_SFPQ      263 GPGGRSEEKISDSEGFKANLSLLRRPGEKTYTQRCRLFVGNLPADITEDEFKRLFAKYGEPGEVFI  328
cons            265 **..*:************************************************************  330

hamster_SFPQ    325 NKGKGFGFIKLESRALAEIAKAELDDTPMRGRQLRVRFATHAAALSVRNLSPYVSNELLEEAFSQF  390
human_SFPQ      329 NKGKGFGFIKLESRALAEIAKAELDDTPMRGRQLRVRFATHAAALSVRNLSPYVSNELLEEAFSQF  394
cons            331 ******************************************************************  396
                                     RRM2
hamster_SFPQ    391 GPIERAVVIVDDRGRSTGKGIVEFASKPAARKAFERCSEGVFLLTTTPRPVIVEPLEQLDDEDGLP  456
human_SFPQ      395 GPIERAVVIVDDRGRSTGKGIVEFASKPAARKAFERCSEGVFLLTTTPRPVIVEPLEQLDDEDGLP  460
cons            397 ******************************************************************  462
                                   NOPS
hamster_SFPQ    457 EKLAQKNPMYQKERETPPRFAQHGTFEYEYSQRWKSLDEMEKQQREQVEKNMKDAKDKLESEMEDA  522
human_SFPQ      461 EKLAQKNPMYQKERETPPRFAQHGTFEYEYSQRWKSLDEMEKQQREQVEKNMKDAKDKLESEMEDA  526
cons            463 ******************************************************************  528
                                           coiled-coil domain
hamster_SFPQ    523 YHEHQANLLRQDLMRRQEELRRMEELHSQEMQKRKEMQLRQEEERRRREEEMMIRQREMEEQMRRQ  588
human_SFPQ      527 YHEHQANLLRQDLMRRQEELRRMEELHNQEMQKRKEMQLRQEEERRRREEEMMIRQREMEEQMRRQ  592
cons            529 ***************************.**************************************  594

hamster_SFPQ    589 REESYSRMGYMDPRERDMRMGGGGTMNMGDPYGSGGQKFPPLGGGGGIGYEANPGVPPATMSGSMM  654
human_SFPQ      593 REESYSRMGYMDPRERDMRMGGGGAMNMGDPYGSGGQKFPPLGGGGGIGYEANPGVPPATMSGSMM  658
cons            595 ************************:*****************************************  660

hamster_SFPQ    655 GSDMV-----------------RMIDVG---------------------  665
human_SFPQ      659 GSDMRTERFGQGGAGPVGGQGPRGMGPGTPAGYGRGREEYEGPNKKPRF  707
cons            661 ****                  * :. *                      709
```

Supplementary Fig. 3: Sequence alignment of hamster and human SFPQ. In the consensus row identity is indicated as (*), low similarity as (.), and high similarity as (:). Hamster and human SFPQ have 98% sequence identity. The alignment was calculated with M-Coffee[1].

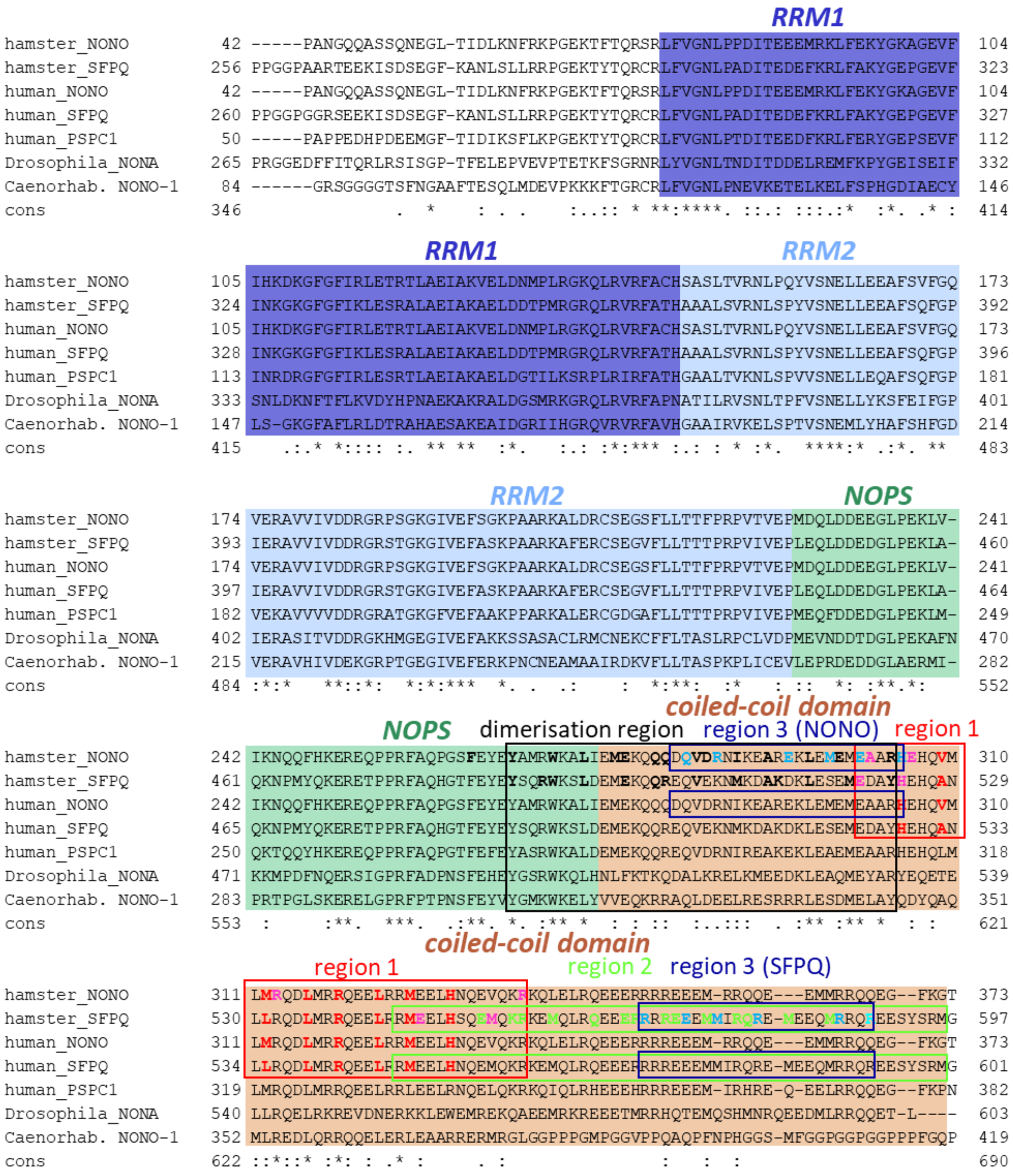

Supplementary Fig. 4: Sequence alignment NONO and SFPQ and contact regions. Sequences of NONO and SFPQ from hamster and human were aligned as well as human PSPC1, NONA from *Drosophila melanogaster* (Uniprot: Q04047)[2] and NONO-1 from *Caenorhabditis elegans* (Uniprot: B3GWA1)[3]. The different domains are indicated by colour shading and binding regions for dimer and filament formation as boxes. Residues involved in helix-helix interactions are marked in bold (see also Supplementary Fig. 14). Dimer formation within the long helix is marked with a black box and letters and was observed in structures of all aligned DBHS proteins shown here. Further interactions for dimer formation in the globular domain are not marked in this figure. A pattern of conserved residues (red letters) in region 1 are involved in binding between NONO and SFPQ in the filaments as well as between SFPQ and SFPQ in crystal structures (PDB: 4WIJ, 4WIK an 6WMZ)[4,5]. There are additional interactions in region 1 that do not follow this symmetric pattern (pink letters). Salt bridges dominate the interhelix contacts in region 2 (green letters). Region 3 interactions are shown in blue letters. R589 from CgSFPQ interacts in region 2 and 3 with another SFPQ and NONO, respectively (cyan letter). In the consensus row identity is indicated as (*), low similarity as (.), and high similarity as (:). The alignment was calculated with M-Coffee[1].

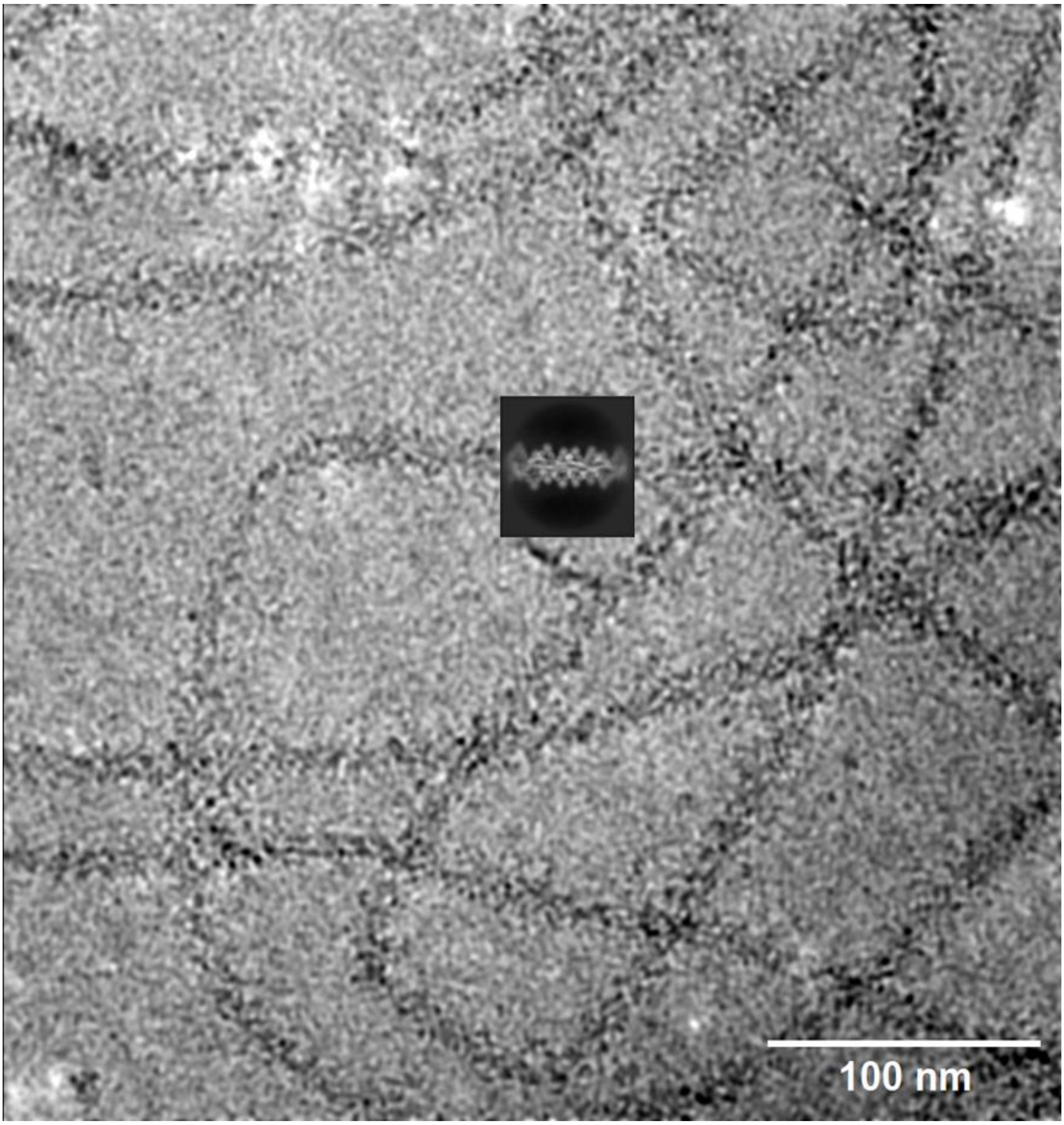

Supplementary Fig. 5: Comparison of filaments on micrographs and a 2D class. One 2D class (Figure 1e) was scaled to the same size as in the micrograph. In both cases the same diameter of about 15 nm is seen. This suggests that the large, disordered N-terminal and C-terminal regions, not resolved in the structure, are not “mantling” closely the filament.

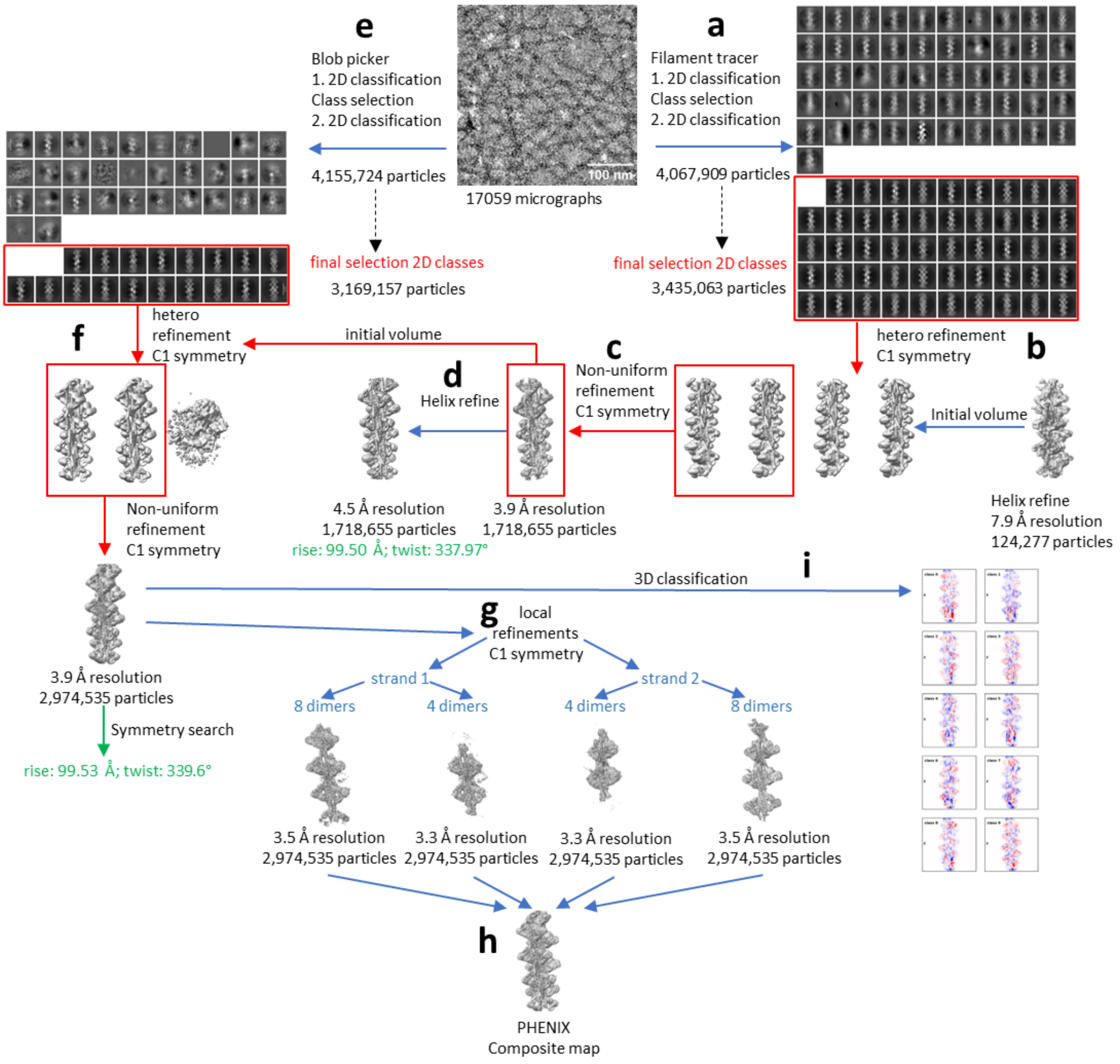

Supplementary Fig. 6: Image processing of NONO/SFPQ filaments. Initially, data were pre-processed in a cryoSPARC[6] live session which included motion correction followed by exposure weighted averaging and patch CTF estimation. All subsequent steps were also performed in cryoSPARC except for the last. **a** Filaments were selected with the filament tracer tool and 4,067,909 segments were extracted. Two rounds of 2D classifications followed to clean up the data set. **b** A helical refinement of an initial data set of 124,277 particles provided a reference volume for a heterogeneous refinement. **c** 2 classes were further refined by non-uniform refinement to a map of 3.9 Å resolution. **d** In a subsequent helical refinement, the resolution decreased to 4.5 Å resolution. **e** An alternative path of data analysis started with the selection of 4,155,725 particles with the blob picker, extraction and clean up in two rounds of 2D classifications, resulting in 3,169,157 particles. **f** A heterogeneous and non-uniform refinement with C1 symmetry led to a map of 3.9 Å resolution. A helical symmetry search provided a rise of 99.53 Å and a twist of 339.6° as best fit. **g** Local refinements of 4 or 8 dimer units on each strand provided best resolved maps of 3.3 or 3.5 Å resolution, respectively. **h** These maps were combined in Phenix[7] to a composite map. **i** A 3D classification demonstrated the flexibility (Supplementary Movie 1), but refinements of classes did not improve resolution.

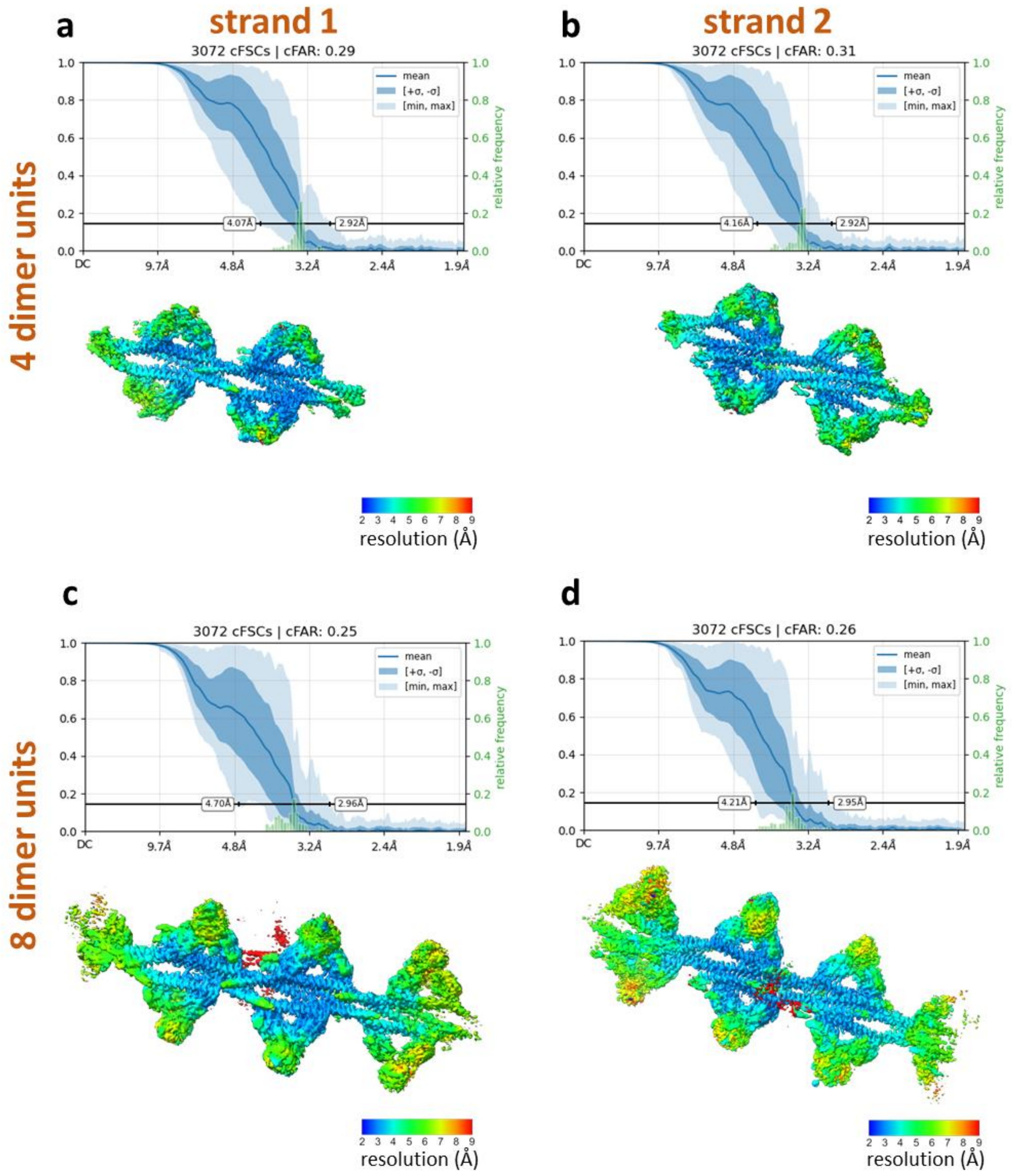

Supplementary Fig. 7: Local refinements of the central units. The directional resolutions (top) and local resolutions (bottom), both calculated with CryoSparc[6], are shown for local refinements of **a** 4 dimer units or **c** 8 dimer units of strand 1. **b** and **d** show the corresponding local refinements of 4 and 8 dimer units of strand 2, respectively.

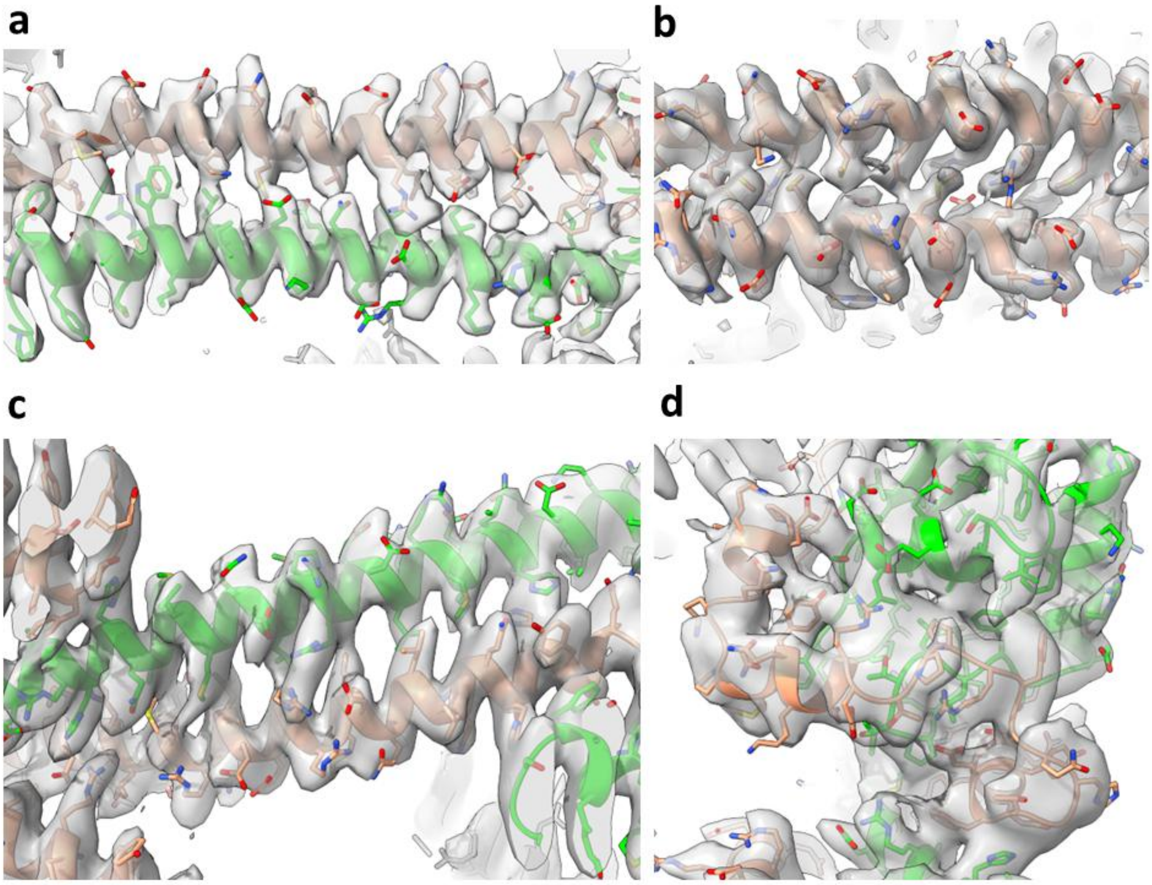

Supplementary Fig. 8: Example densities and models of strand 2. NONO is coloured green and SFPQ brown: **a** dimerization domain, **b** region 2, **c** region 1, and **d** RRM2 and NOPS. The map is shown at a contour level of 10.

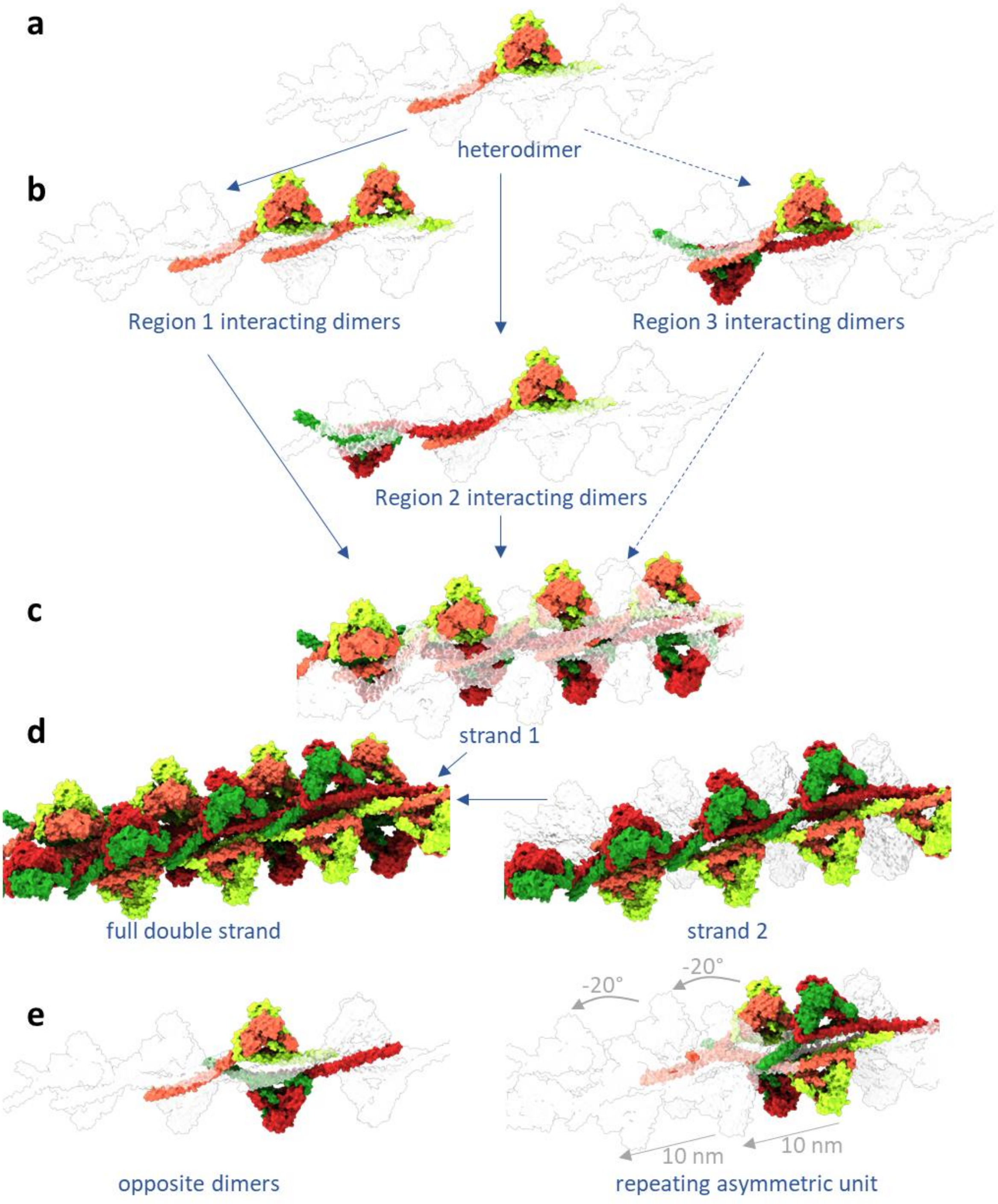

Supplementary Fig. 9: Organisation of the filament. a The obligate heterodimer with many interactions in the globular domain is the starting point of oligomerisation. **b** Substantial interactions between different dimers are provided by the coiled-coils in regions 1 and 2 while region 3 provides only small contributions. **c** These interactions built up a complete strand. **d** Two strands form the final filament. **e** Opposite dimers on the same strand have no direct contact. Two of these tetramers represent the repeating asymmetric unit of the filament, assuming an idealised symmetric structure.

a

#	AA	Sequence of automatic modelling with ModelAngelo	BLAST	Assign-ment
1	135	ESKAEECRSVPPKFAQKKGTFEYERAQRWKDLDEEEKQQRNQTEKQLKEAQE KIESNQKAAYFEYQVNLLRNELKRRKAELKRLEELYEELFQKRQAFQERQEQER RRQEEKQMNHKRKLEEHFKKNREASGKAG	SFPQ	SFPQ
2	128	SREPTWAQKGTYEWERSQRYLSLDEEEKKMYKQVSENERDAKEKLSSNQEAA YHNYNAQLLRNEIEKRNEQLRKLEEEFDQCQQKRKEQQERQEEERKRRLEEF LLKQREMMSQQKKLRSVLEAKFRR	SFPQ	SFPQ
3	96	TCAGKPGSFEHERAMRWMALIEQEKNQQDQVDREVKEAREKNELEQLAARY EYQVEEMRKEVNQYQEELRHLKDNFKQEENKNRREEKEEKEKEEE	NONO	NONO
4	86	RQKKLAADPPRRSNDGGSFEWEWAMRYKSLTEQKKQKQEQVSRNVKEARKK LEMEQEAARYQYQVELKRKLINRYKEELKRLMEEG	NONO	NONO
5	82	LENERAIRWIEVLSELQNQKEQVSRNVKKARDQLKLEQPAARYNWKVELMKK EVERRQEELKRLKAEFNKELNQRKEEEKKK	NONO	NONO
6	76	DKPVFRADPDGGFEYERAMRWKAIIEQKKQQQSQVERNIKEAREKLKMELEA ARHTYQIQLLKQEIERNKAAALFK	NONO	NONO
7	73	NEHEFEEQEESLQRTQEELRRLEELFEQMMQKRQEFQNRMEEERRRREEEQ MIHKREQMSQLREKREEASASL	not found	SFPQ
8	67	GTGQEHERSQRWKSLDEEVKEKYEKLEQQMQAEESKIESQGEKAAYYEFEAN ELKQEAYKERYLEEQ	SFPQ	SFPQ
9	64	SFEHERAQRWKSLDEEKKQQYNQVEQQLKAAEEKLLDLREADYHEQNANEL KQEMKKKKEELVK	SFPQ	SFPQ
10	53	QKEEFEKCMQQRKLFQNRLEEERRRREEDMQIKKYEFLNKIHKKKSVSFFLSR	not found	SFPQ

b

#	AA	Sequence of automatic modelling with ModelAngelo	BLAST	Assign-ment
1	124	PPRRAEQGTFERERAQRWKSLDEEEKKKYNQIEKQEKSAKEKLEALLEVAHYE HNVQNLKQLLEKRQEELRKLEELFEQRQQERKDLQERLEEERRREEENQLER MRTMMEHFKRNRSVAVFGK	SFPQ	SFPQ
2	88	GGFKRDFAKRWLDLLENENQNQSQVTRQIQEAREELELKKAAARYEHQVEL MKQELQKYKKEERQLKSTFKREEEENRKEKEEEEEKE	NONO	NONO
3	82	KLYLGNDGKTRASPGGFEYEWAKRWKALQEQQQKQQEQVDRNVKEAREKL ELLMDAARFEYKVKNLKEESLEERLEEEALEL	NONO	NONO
4	78	RKREYELENQLLSLERTNDSLKKLELLFEQKQQERKEMKNRFEEERRHREEEM MIRKREFIVLLRHFKELLLKKALRK	not found	SFPQ
5	77	ARHEYQAELLRDEIERRQAELRFLEEKYEECQQKRQDFQNRNETERKRRLTEK QNNQRKMMEFQKRNRELLERLLGK	not found	SFPQ
6	76	RALNEEKKNQQEQVSRNIKEARLKLEMDELAARYEHQVELQRQEVERYKEEL KHLLANFKREEQKRRKEQEEAKER	NONO	NONO
7	75	PFRSKPGSFTHERAERWQAITEEEKQQQSQVERNIKEAREKLELEQEAARYEY QVQMKRQELQRRKEEMKQLEAS	NONO	NONO
8	73	EAEELIRETTKRFQELKHMENNFEQCQQKTKIQQNRKEEVRRKRMELMQIKQ RELMNQLRKLRASLESGLLLK	not found	SFPQ
9	49	PPSRAEKGTYKYEYANRWKDLDEMSQNQREKVEKRMKDEEAKLLEEGEE	SFPQ	SFPQ
10	48	DPTRYEKGVFDWLRSMKWQSLEEEEKQMRKQVSKNLKSAKEKLESNQK	SFPQ	SFPQ

Supplementary Fig. 10: Assignment of subunit identity by automatic modelling. The highest resolution local refinements of four dimer units of **a** stand 1 and **b** strand 2 were subjected to automatic modelling with the program ModelAngelo[8]. The ten longest fragments of the modelling for each strand were then searched with BLAST[9] and it was evaluated if NONO or SFPQ scored higher (second last column). Some fragments did not give a significant similarity with the standard settings of BLAST. The last column shows the assignment in our models.

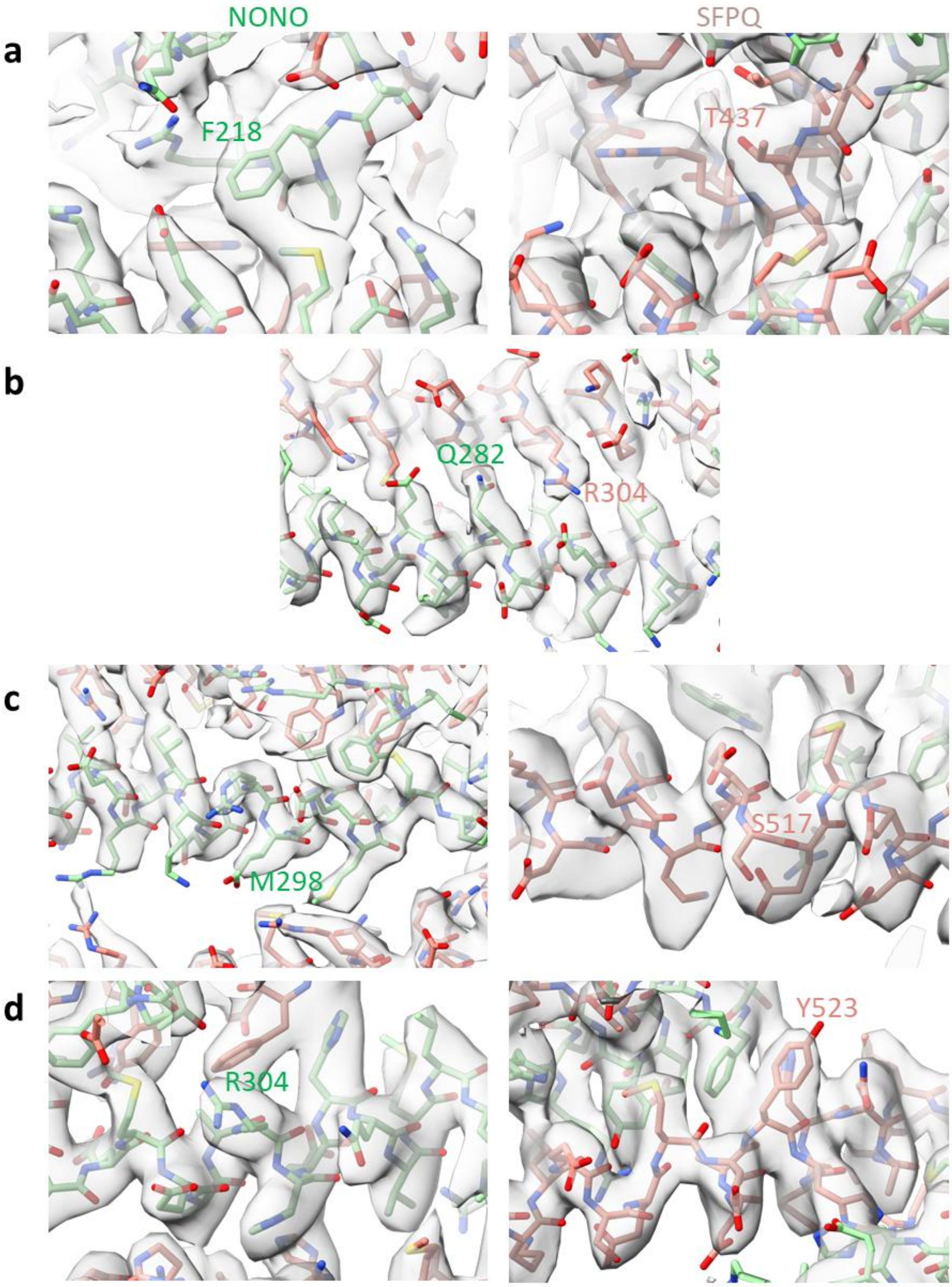

Supplementary Fig. 11: Assignment of subunit identity at specific locations. Based on the sequence alignment specific equivalent positions can be distinguished by the side chain densities. Four pairs are represented here with NONO on the left side and SFPQ on the right. **a** Side chain density of F218 in NONO is considerably larger than the corresponding T437 in SFPQ. **b** Q282 in NONO is shorter than R501 in SFPQ. **c** M298 in NONO and S517 in SFPQ **d** R304 in NONO and Y523 in SFPQ.

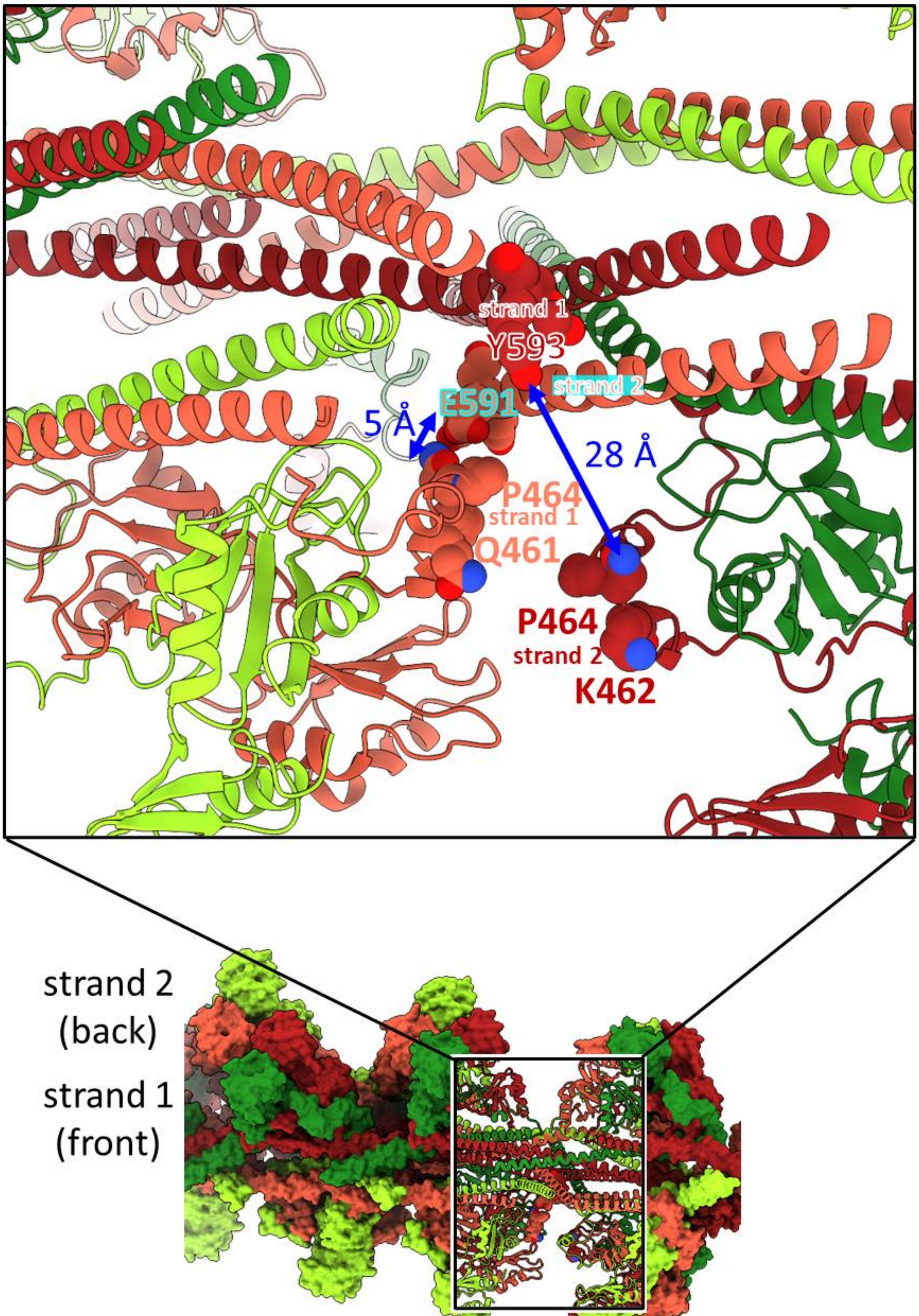

Supplementary Fig. 12: Interstrand contact point. The end of the long α-helix of SFPQ (R591-A594) is shown as spheres as well as the contact loop of the NOPS domain (Q461-P464). For the light red SFPQ subunits the interstrand contact is established but not for the dark red SFPQ pair. Here the contact is blocked by the long helix of the light red SFPQ (cyan labelling). Colouring as in Figure 1f. For the zoom in only a layer is shown so that the contacts are visible.

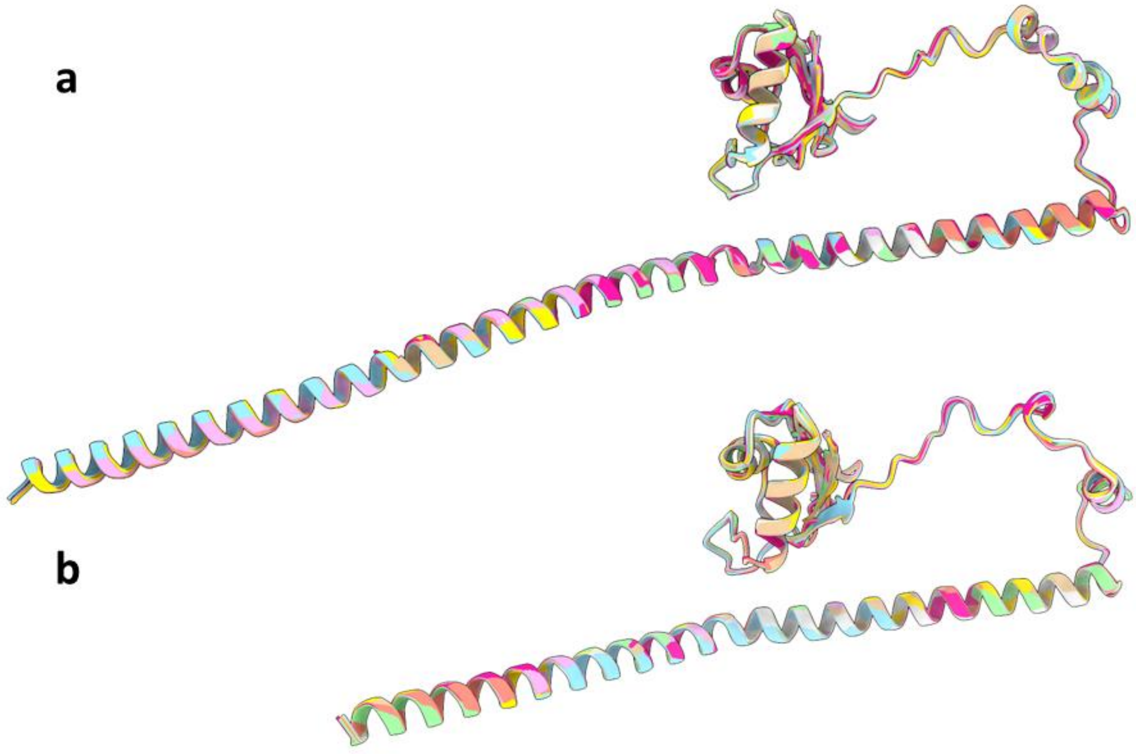

Supplementary Fig. 13: Overlay of subunits from the local refinements. Using ChimeraX[10] matchmaker, subunits of **a** SFPQ or **b** NONO from models of strand 1 and 2 were overlayed. No significant differences in the backbone can be seen.

a Dimerisation region

CfSFPQ	Homologue residue in hSFPQ	CgNONO (and hNONO)	Binding interaction
**F482**	F486	**R304**	Hydrophobic interaction
**Y486**	Y490	**L296**	H-bond
**R489**	R493	**E299**	Salt bridge
**W490**	W494	**L296**	Hydrophobic interaction
**L493**	L497	**A292**<br>**I289**	Hydrophobic interaction<br>Hydrophobic interaction
**E497**	E501	**I289**	Hydrophobic interaction
**Q500**	Q504	**V285**	Hydrophobic interaction
**R501**	R505	**D286**	Salt bridge
**V504**	V509	**V285**<br>**Q282**<br>**Q281**	Hydrophobic interaction<br>Hydrophobic interaction<br>Hydrophobic interaction
**M508**	M512	**E278**<br>**M277**	Hydrophobic interaction<br>Hydrophobic interaction
**A511**	A515	**L274**	Hydrophobic interaction
**K512**	K516	**E278**	Salt bridge
**L515**	L519	**W271**<br>**Y267**	Hydrophobic interaction<br>H-bond
**M519**	M523	**Y267**	Hydrophobic interaction
**Y523**	Y527	**F263**	Hydrophobic interaction

b Region 1

CfSFPQ	Homologue residue in hSFPQ	CgNONO (and hNONO)	Binding interaction
**E520**	E524	**R337**	Salt bridge
**H524**	H528	**H330**	π-π stack, cation-π interaction
**A528**	A532	**M326**	hydrophobic interaction
**L531**	L535	**M326**<br>**L323**	hydrophobic interaction<br>hydrophobic interaction
**L535**	L539	**L323**<br>**L316**	hydrophobic interaction<br>hydrophobic interaction
**R538**	R542	**R319**	Arg-Arg bridge
**L542**	L546	**L316**<br>**M312**<br>**V309**	hydrophobic interaction<br>hydrophobic interaction<br>hydrophobic interaction
**M545**	M549	**M312**<br>**V309**	hydrophobic interaction<br>hydrophobic interaction
**E546**	E550	**R313**	Salt bridge
**H549**	H553	**E306**	H-bond, salt bridge, no interaction to H305
**M553**	M557	**A302**	hydrophobic interaction

c Region 2

CfSFPQ	Homologue residue hSFPQ	CgSFPQ	Binding interaction
**E552**	E556	**R589**	Salt bridge
**K555**	K559	**M585**<br>**M581**	Hydrophobic interaction
**R556**	R560	**E582**	Salt bridge
**M559**	M563	**M581**	Hydrophobic interaction
**Q563**	Q567	**M574**	H-bond (CO); hydrophobic interaction
**E566**	E570	**R570**<br>**R577**	Salt bridge<br>Salt bridge
**R567**	R571	**E571**	Salt bridge
**R570**	R574	**R570**<br>**E571**	Hydrophobic interaction<br>Salt bridge
**E571**	E575	**R567**	Salt bridge
**M574**	M578	**Q563**	H-bond (CO); hydrophobic interaction
**R577**	R581	**E566**	Salt bridge
**Q578**	Q582	**Q560**	H-bond
**M581**	M585	**M559**	Hydrophobic interaction
**M585**	M589	**K555**	Hydrophobic interaction
**R589**	R593	**E552**	Salt bridge

d Region 3

CfSFPQ	Homologue residue hSFPQ	CgNONO (and hNONO)	Binding interaction
**R568**	R572	**Q284**<br>***or* R287**	H-bond<br>Arg-Arg bridge
**E572**	E576	**R287**	Salt bridge
**M575**	M579	**M298**	Hydrophobic interaction
**R579**	R583	**E294**<br>**M298**	Salt bridge<br>Hydrophobic interaction
**R586**	R589	**E301**	Salt bridge
**R589**	R593	**H305**	cation-π interaction

Supplementary Fig. 14: Interactions between different subunits in the contact regions of the long helices. The different interactions are listed for **a** the dimerisation region, **b** region 1, **c** region 2, and **d** region 3. The colouring is the same as the bold letters in the sequence alignment of Supplementary Fig. 4.

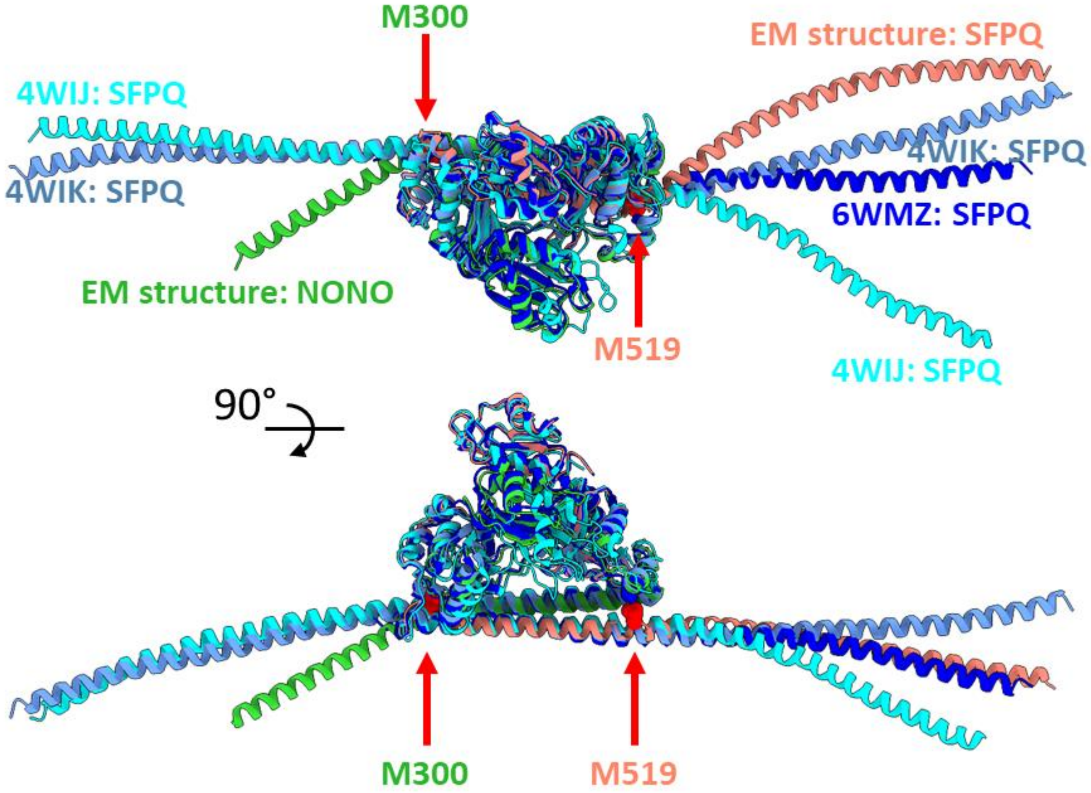

Supplementary Fig. 15: Kink in the long α-helix: SFPQ shows a pronounced kink (red arrows) at M519 in the filamentous structure (salmon) which is only small in the crystal structures of SFPQ (PDB: 4WIJ, 4WIK; cyan and cornflower blue)[4] and NONO/SFPQ (PDB: 6WMZ; inkblue)[5]. NONO displays a similar kink at residue M300 in the filamentous structure (green). No crystal structure of the extended α-helix beyond the globular domain of NONO is available.

a

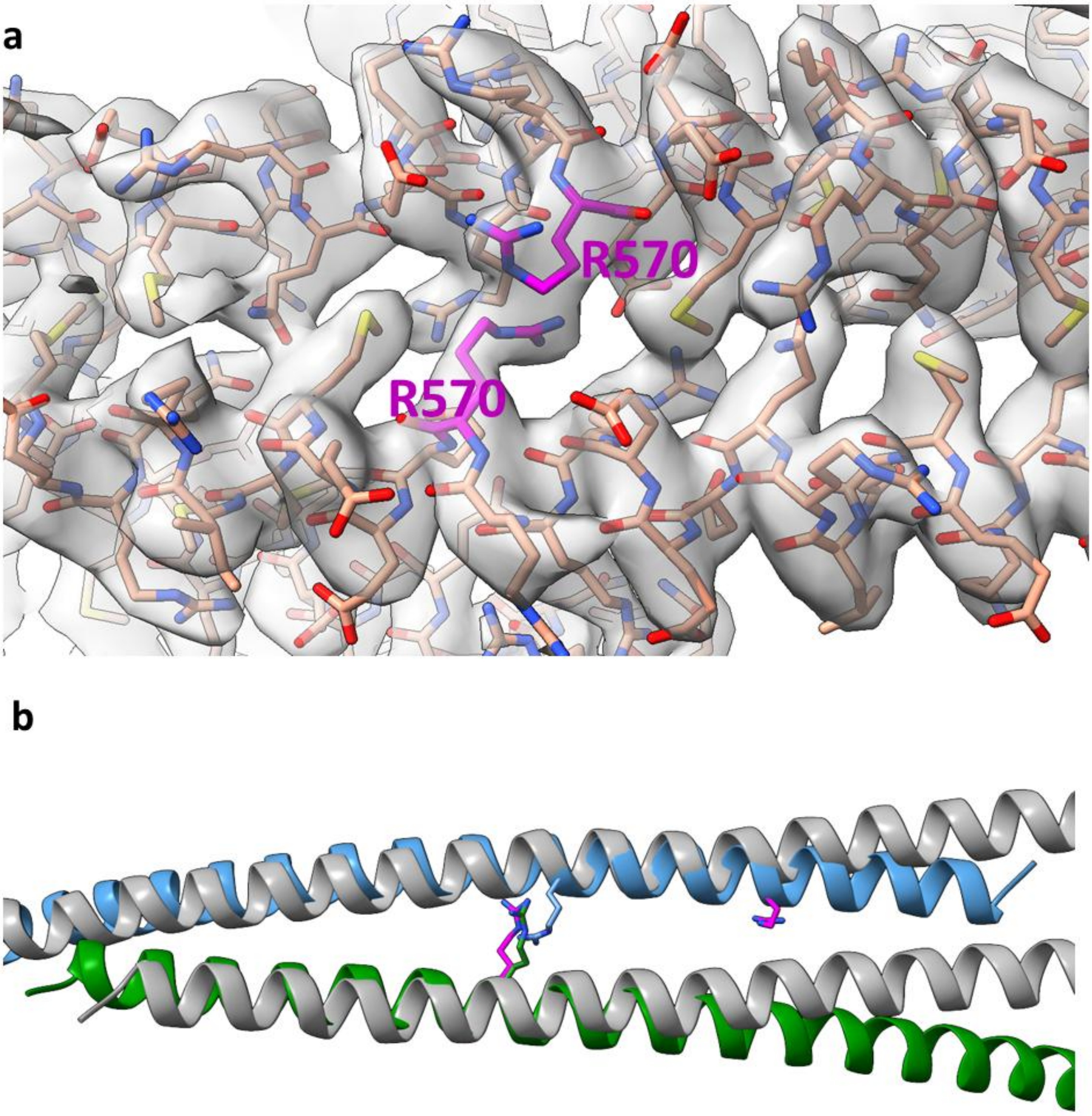

b

Supplementary Fig. 16: Coiled-coil region 2: a Densities and models of region 2 is shown with the central R570 (pink) where the same residues of the different subunits meet. The densities suggest that no arginine-arginine bridge is formed and the side chains face in different directions. Consequently, they have a different conformation in these non-equivalent SFPQ subunits (in terms of helical symmetry). **b** overlay of region 2 from the filament (coloured helices) and a SFPQ crystal structure (grey, PDB: 4WIK)[4]. Side chains of R570 (CgSFPQ, green and blue) and the homolog R574 (human SFPQ, pink) are shown which indicate that the helices are shifted by 3 turns and also have different degrees of bending.

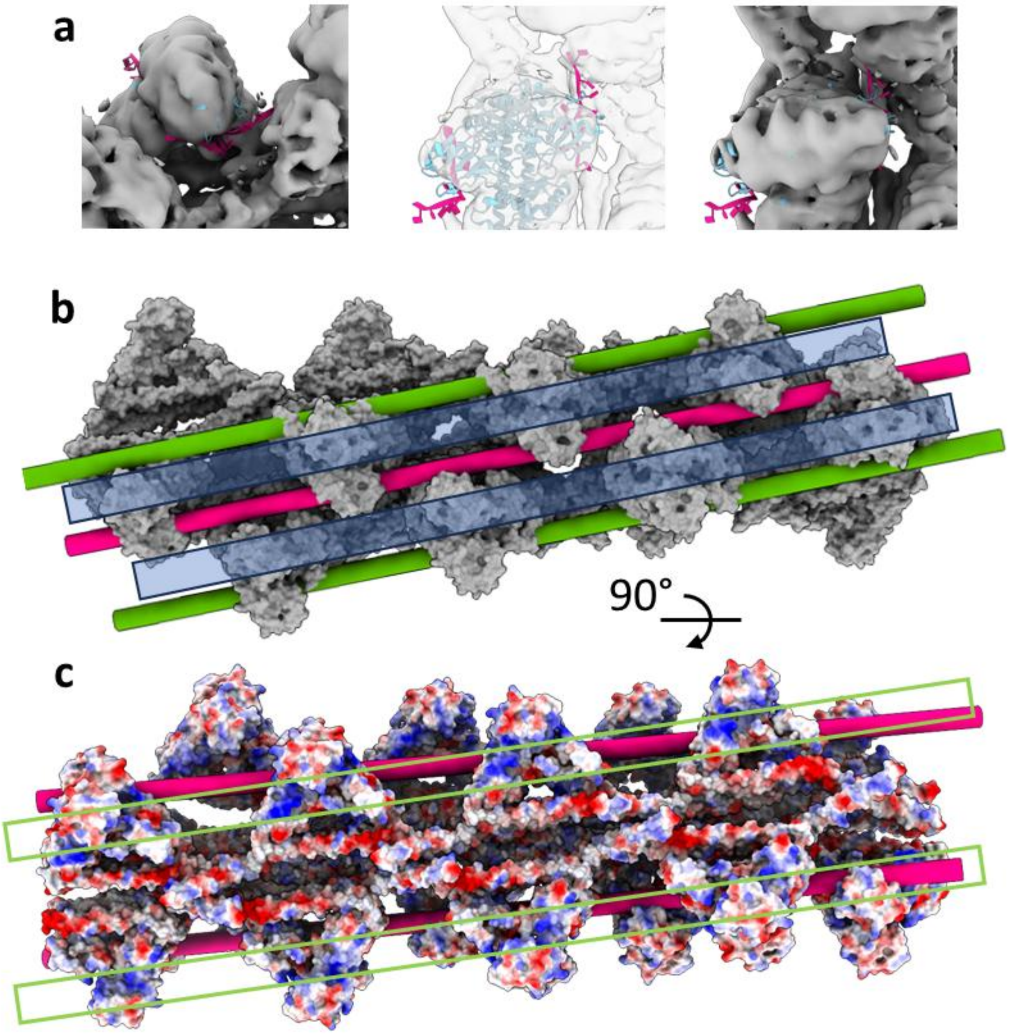

Supplementary Fig. 17: Possible interactions of RNA and DNA with the filaments. a The crystal structure of the SFPQ homodimer shows the interaction with RNA between RRM1 and 2 on each subunit (PDB 7UJ1)[11]. This crystal structure (shown as model) was rigidly fitted into the globular domain of the EM filament map. This would place the RNA (pink) bound to one subunit into the groove between the strands. The other bound RNA on the other subunit faces outwards. An image with solid and with transparent EM density is shown and a different view. **b** Surface representation of NONO/SFPQ model (grey). RNA (pink) could be locked in between the two strands interacting with the binding sites of SFPQ which have opposite directions on both strands. Alternatively, RNA could bind on the periphery to the binding sites of NONO (green). The unresolved DNA binding domain of SFPQ is N-terminal to RRM1 and thus probably close to the top of the heads (blue box). **c** Electrostatic surface representation of the model viewed onto one strand (about 90° to view in **b**). Positively charged grooves of NONO continuing from one head to the next is the likely location for RNA binding (green boxes).

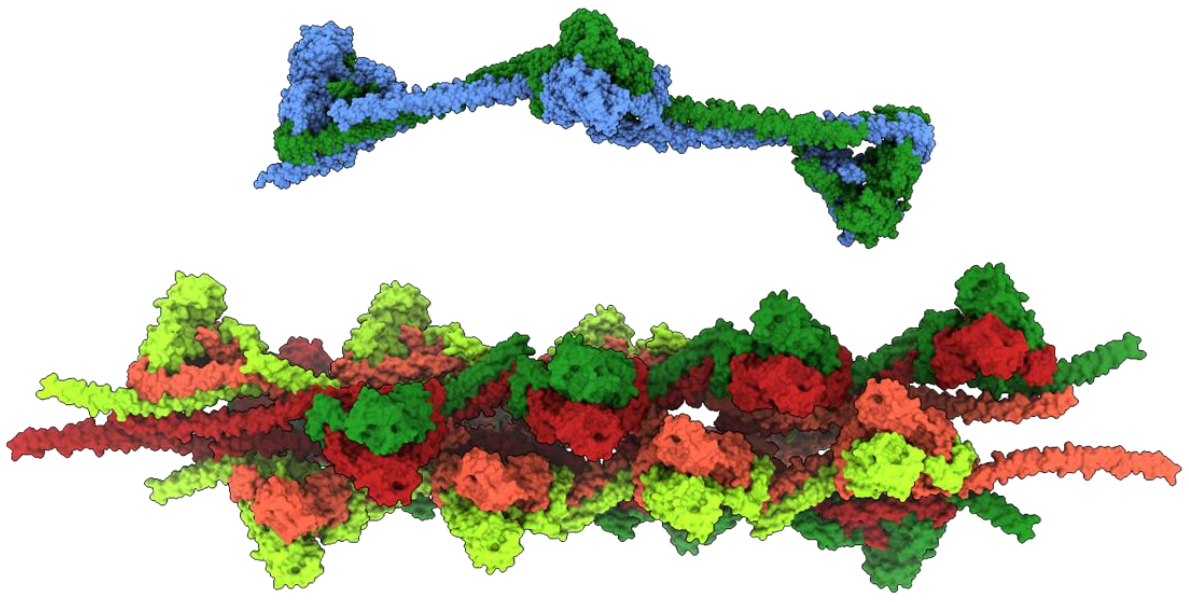

Supplementary Fig. 18: Comparison of filamentous models. The filamentous *in silico* model of NONO/PSPC1 from Dobson et al.[12] (top) is compared with our filaments (bottom). SFPQ is coloured in red, NONO in green and PSPC1 in blue. The distance between the dimer globular domains and the twist is smaller in our filaments.

a   b

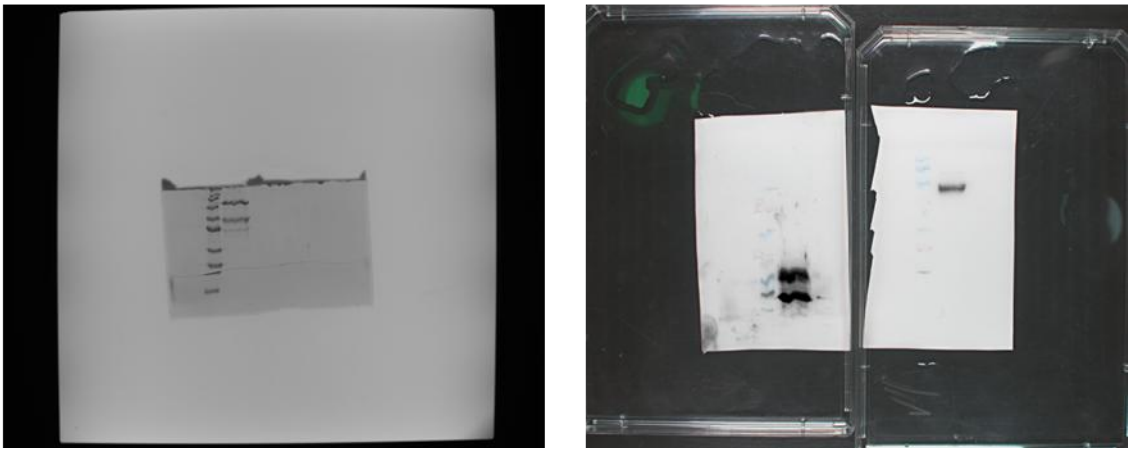

Supplementary Fig. 19: Uncropped SDS-PAGE and Western Blot. a The uncropped images of the Coomassie-stained SDS-PAGE and **b** Western blot (right blot) from Supplementary Fig. 1b are presented here.

Supplementary References:

1. Di Tommaso, P. *et al.* T-Coffee: A web server for the multiple sequence alignment of protein and RNA sequences using structural information and homology extension. *Nucleic Acids Res.* **39**, W13–W17 (2011).

2. Rendahl, K. G., Gaukhshteyn, N., Wheeler, D. A., Fry, T. A. & Hall, J. C. Defects in courtship and vision caused by amino acid substitutions in a putative RNA-binding protein encoded by the no-on-transient A (nonA) gene of Drosophila. *J. Neurosci.* **16**, 1511–1522 (1996).

3. Knott, G. J., Lee, M., Passon, D. M., Fox, A. H. & Bond, C. S. Caenorhabditis elegans NONO-1: Insights into DBHS protein structure, architecture, and function. *Protein Sci.* **24**, 2033–2043 (2015).

4. Lee, M. *et al.* The structure of human SFPQ reveals a coiled-coil mediated polymer essential for functional aggregation in gene regulation. *Nucleic Acids Res.* **43**, 3826–3840 (2015).

5. Koning, H. J. *et al.* Structural plasticity of the coiled–coil interactions in human SFPQ. *Nucleic Acids Res.* **53**, gkae1198 (2025).

6. Punjani, A., Rubinstein, J. L., Fleet, D. J. & Brubaker, M. A. cryoSPARC: algorithms for rapid unsupervised cryo-EM structure determination. *Nat. Methods 2017 143* **14**, 290–296 (2017).

7. Adams, P. D. *et al.* PHENIX: A comprehensive Python-based system for macromolecular structure solution. *Acta Crystallogr. Sect. D Biol. Crystallogr.* **66**, 213–221 (2010).

8. Jamali, K. *et al.* Automated model building and protein identification in cryo-EM maps. *Nature* **628**, 450–457 (2024).

9. Altschul, S. F., Gish, W., Miller, W., Myers, E. W. & Lipman, D. J. Basic local alignment search tool. *J. Mol. Biol.* **215**, 403–410 (1990).

10. Pettersen, E. F. *et al.* UCSF ChimeraX : Structure visualization for researchers, educators, and developers. *Protein Sci.* **30**, 70–82 (2021).

11. Wang, J. *et al.* Insight into the Tumor Suppression Mechanism from the Structure of Human Polypyrimidine Splicing Factor (PSF/SFPQ) Complexed with a 30mer RNA from Murine Virus-like 30S Transcript-1. *Biochemistry* **61**, 1723–1734 (2022).

12. Dobson, L., Nyitray, L. & Gáspári, Z. A conserved charged single α-helix with a putative steric role in paraspeckle formation. *RNA* **21**, 2023–2029 (2015).
