# Supplementary material for: The gene-regulating proteins NONO and SFPQ assemble into ordered filaments
Source: Commun Biol. 2025 Dec 31;9:117. doi: 10.1038/s42003-025-09396-8 (PMC12848012; doi:10.1038/s42003-025-09396-8)
Supplement: Supplementary file 3 — Description of Additional Supplementary Files [file 42003_2025_9396_MOESM3_ESM.pdf]

Description of Additional Supplementary File

File name: Supplementary Movie 1
Description: 10 Classes of the 3D classification shown as a sequence of images.

File name: Supplementary Data
Description: Size Exclusion Chromatography and Dynamic Light Scattering traces of Supplementary Fig. 1 are provided as numerical data.
